# *NBEAL2* gene mutations do not always lead to gray platelet syndrome: A case report

**DOI:** 10.1097/MD.0000000000039975

**Published:** 2024-10-04

**Authors:** Bing Chen, Wanzhong Kong, Jinlin Liu, Junwu Zhang

**Affiliations:** a Department of Clinical Laboratory, Wenchang People’s Hospital, Wenchang, Hainan, China; b Department of Clinical Laboratory, Wenzhou TCM Hospital of Zhejiang Chinese Medical University, Wenzhou, China; c Department of Clinical Laboratory, South China Hospital, Medical School, Shenzhen University, Shenzhen, China.

**Keywords:** gray platelet syndrome, nonsense *NBEAL2* gene mutation

## Abstract

**Rationale:**

Gray platelet syndrome (GPS) is a rare disease caused by homozygosity and compound heterozygosity for autosomal mutations on the *NBEAL2* gene, which is characterized by a deficiency of platelet α-granules, bleeding symptoms. However, in this study, we report 2 *NBEAL2* gene mutations in an easy bruising family without gray platelet and bleeding.

**Patient concerns:**

A 33-year-old female nurse sought admission to our laboratory due to a tendency to bruise easily and unwell in daily life. However, there are no signs of petechiae or excessive bleeding in her daily life. Coagulation tests, routine blood tests and platelet staining of blood smears were all normal.

**Interventions:**

The whole exome sequencing and Sanger sequencing were used to identify the causative variant of the patient. Furthermore, the morphology of platelets was examined using electron microscopy.

**Diagnosis and outcomes:**

Whole exome sequencing revealed the presence of 2 mutations in the *NBEAL2* gene: p.Thr365fs and p.Ala310Thr. This prompted the consideration of a GPS diagnosis. However, platelet electron microscopy did not identify any abnormalities, leading to the exclusion of GPS.

**Lessons:**

These 2 *NBEAL2* gene mutations (p.Thr365fs and p.Ala310Thr mutations) do not affect the degranulation of platelets.

## 1. Introduction

Homozygosity/compound heterozygosity for loss of function mutations in neurobeachin-like 2 (*NBEAL2*) leads to gray platelet syndrome (GPS), which is characterized by thrombocytopenia and large platelets lacking α-granules and cargo.^[1]^ Recently, novel clinical phenotypes were also observed, including reduced leukocyte counts, increased presence of autoimmune diseases, positive autoantibodies, and the immunity goes awry beyond the bleeding disorders in GPS.^[2,3]^ *NBEAL2* plays a crucial part in the production, development, and functionality of platelets, and deficiency could lead to mild to moderate bleeding.^[4]^ However, in this study, we report 2 *NBEAL2* gene mutations in an easy bruising family without gray platelet, which further corroborate that these 2 *NBEAL2* mutations could generate nonsense codons, and not all NBEAL2 mutations lead to GPS.

## 2. Case presentation

In this study, the patients were anonymized. They provided informed consent for the publication of their cases. This study has been approved by the ethical committee of Wenzhou TCM Hospital of Zhejiang Chinese Medical University.

**Figure 1.**
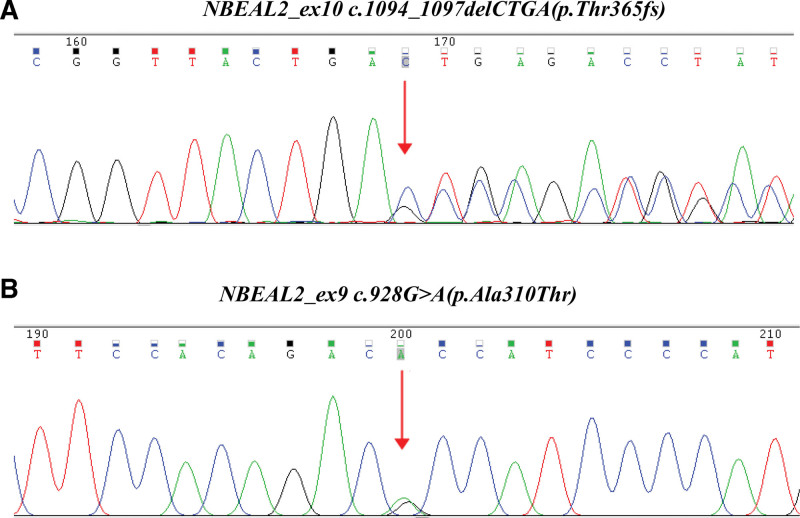
Sanger sequencing analysis of this female nurse. The Sanger sequencing analysis identified 2 mutations in this female nurse: (A) *NBEAL2*_ex10 c.1094_1097delCTGA(p.Thr365fs) and (B) *NBEAL2*_ex9c.928G>A(p.Ala310Thr).

**Figure 2.**
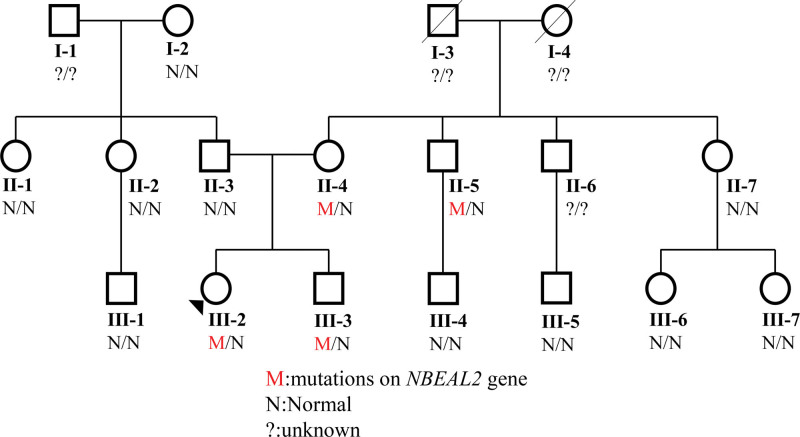
Family pedigree. The proband is indicated by the black triangle. Family members who did not undergo genetic testing were classified as unknown.

**Figure 3.**
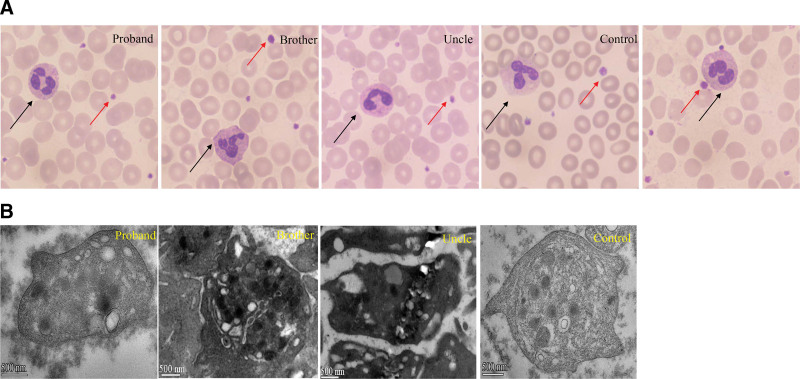
Neutrophils and platelets with normal morphology in this family members. (A) Neutrophils (black arrows) and platelets (red arrows) with normal morphology were observed (magnification, 1000×; Wright–Giemsa staining). (B) The ultrastructure of platelets with a transmission electron microscope in these family members and healthy control.

**Figure 4.**
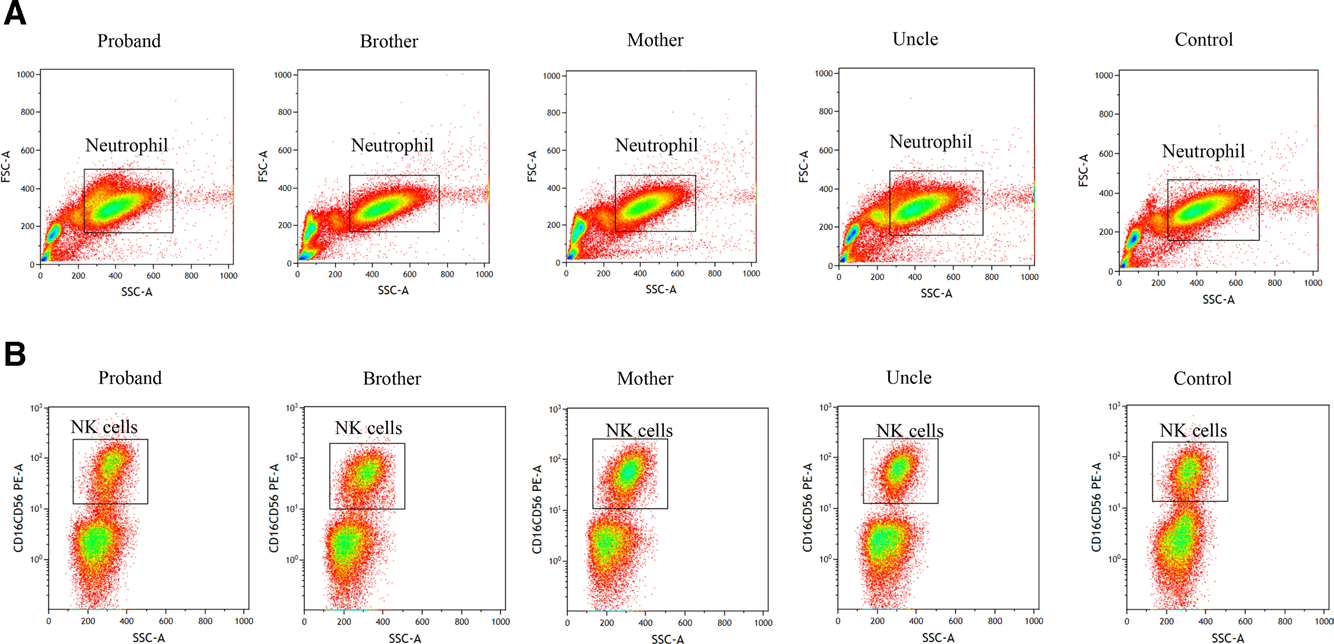
The degranulation of neutrophils and NK cells was not significant change in these family members. (A) Scatter plots of neutrophils. (B) Scatter plot of CD16^+^CD56^+^NK cells.

A 33-year-old female nurse was admitted to our laboratory to ask for help for an accurate diagnosis of her being unwell in daily life. Upon further interrogation, she was easily bruised but denied having petechiae and no excessive bleeding in daily life. Additionally, this patient had no family history of coagulation disorder or other hematological disorders. Coagulation tests including PT, INR, APTT, TT, and Fib indices were normal. Platelet count and platelet aggregation rate with adenosine diphosphate and arachidonic acid were all within normal range, did not support coagulation or platelet disorders. However, the exact cause of unwell and prone to easy bruising remains mysterious. Then the blood sample from this patient was sent to the reference laboratory for a whole exome sequencing to find the cause. One month later, the genetic tests revealed that she had 2 p.Thr365fs and p.Ala310Thr mutations on the *NBEAL2* gene, which were validated by Sanger sequencing (Fig. 1A and B), the GPS diagnosis was then suspected. Moreover, the blood samples of all her family members were also sent for genetic tests, which revealed her mother, uncle and brother had the same *NBEAL2* mutations validated by Sanger sequencing (Fig. 2). Upon interrogation, all these family members were easy to bruise in daily life, but denied having petechiae, excessive bleeding and being unwell in daily life. Moreover, the peripheral blood smear of this patient and other family members with *NBEAL2* gene mutations were reexamined whether the platelets or neutrophils were gray. In contrast, the platelets or neutrophils were not gray, neither degranulation nor aggregation was observed on the blood smear (Fig. 3A). This prompted us to further examine the ultrastructure of platelets by the method of the transmission electron microscope, but no significant changes were observed compared to the health individual (Fig. 3B). Unfortunately, her mother refused to electron microscope examination. Together, we report these 2 p.Thr365fs and p.Ala310Thr *NBEAL2* mutations could generate nonsense codons in this family. Moreover, novel clinical phenotypes were also observed which the immunity defects beyond the bleeding disorders in GPS, and *NBEAL2* mutation was also required for neutrophil and NK cell function.^[5]^ Thus, neutrophils and NK cells were both examined by the flow cytometry to examine the degranulation in this family, but no significant results were observed in these family members(Fig. 4A and B), demonstrating that these 2 *NBEAL*2 mutations were not associated with the degranulation of neutrophil or NK cells. At the subsequent follow-up visit in August 2024, the patient and family members reported no symptoms apart from easy bruising in daily life.

## 3. Discussion

GPS is a rare recessive disorder caused by biallelic variants in *NBEAL2* and characterized by bleeding symptoms, the absence of platelet α-granules, splenomegaly, and bone marrow fibrosis.^[6,7]^ Like GPS patients, mice lacking *NBEAL2* expression exhibit macrothrombocytopenia, deficiency of platelet α-granules, splenomegaly, myelofibrosis, impaired platelet function and abnormalities in megakaryocyte development.^[1]^ However, we present a family with 2 novel *NBEAL2* gene mutations but the platelet was not gray, and α-granules of platelet were also not deficient.

In addition to the well-described platelet defects in GPS, immune defects were also reported, including reduced leukocyte counts, increased presence of autoimmune diseases, positive autoantibodies, and the immunity goes awry beyond the bleeding disorders in GPS.^[2,3]^ Drouin et al^[8]^ first described not only gray platelets but also gray polymorphonuclear neutrophils with decreased or abnormally distributed components of secretory compartments in GPS patients. Chedani et al^[9]^ also revealed that neutrophils had the secretory defects that existed in GPS patients. Moreover, these clinical phenotypes were summarized in a large cohort of 47 GPS patients, including reduced leukocyte counts, increased presence of autoimmune disease and positive autoantibodies, the multiple types of blood cells are deficient in granule proteins.^[2,3]^ Moreover, John et al^[5]^ found that *NBEAL2* is required for neutrophil and NK cell function and pathogen defense in *NBEAL2*-deficient mice, neutrophils showed a severe reduction in granule contents across all granule subsets, and the *NBEAL2*-deficient NK cells were dysfunctional and showed reduced degranulation. However, this study also demonstrated that these 2 novel *NBEAL2* mutations were not associated with the degranulation of neutrophil or NK cells.

*NBEAL2* is the major source of mutations in GPS that have long been considered a classic inherited platelet disorder resulting from a lack of α-granules and their contents.^[10]^ Herein, 2 *NBEAL2* mutations in this family were found by accident while the genetic results revealed 2 *NBEAL2* gene mutations. *NBEAL2*_ex10 c.1094_1097delCTGA(p.Thr365fs), a heterozygous frameshift mutation, which resulted in the change of threonine to serine at 365 amino acid position and produces a new reading frame. Another heterozygous missense mutation in the *NBEAL2* gene [*NBEAL2*_ex9c.928G>A(p.Ala310Thr)] was also found. However, the patient had normal platelet morphology and related tests and only bruised easily in daily life. It can be inferred that a single null allele may not be sufficient to cause GPS (homozygous or compound heterozygous variants are needed). Nevertheless, it is conceivable that a mild phenotype, such as easy bruising, may also be observed. However, this study had several limitations. Firstly, no comprehensive functional testing of platelets was conducted on the patients and their families. Secondly, a long-term follow-up of patient and their families was not conducted.

## 4. Conclusion

To the best of our knowledge, these 2 mutations had not been reported, and these 2 *NBEAL2* gene mutations (p.Thr365fs and p.Ala310Thr mutations) do not affect the degranulation of platelet.

## Author contributions

**Conceptualization:** Jinlin Liu, Wanzhong Kong, Junwu Zhang, Bing Chen.

**Data curation:** Bing Chen, Wanzhong Kong.

**Formal analysis:** Bing Chen, Wanzhong Kong, Junwu Zhang.

**Resources:** Junwu Zhang, Wanzhong Kong.

**Writing – original draft:** Jinlin Liu, Junwu Zhang.
